# Assessing Oxidative Stress by Thiol/Disulfide Homeostasis Among Vitamin D-Deficient Patients

**DOI:** 10.7759/cureus.20400

**Published:** 2021-12-13

**Authors:** Emin Gemcioglu, Salih Baser, Nuray Yilmaz Cakmak, Özcan Erel, Büsra Tugce Akman, Parvana Ahmadova, Osman Ersoy

**Affiliations:** 1 Department of Internal Medicine, Ankara City Hospital, Ankara, TUR; 2 Department of Internal Medicine, Ankara Yıldırım Beyazıt University, Faculty of Medicine, Ankara, TUR; 3 Department of Biochemistry, Ankara Yıldırım Beyazit University, Faculty of Medicine, Ankara, TUR; 4 Department of Hematology, Baskent University Faculty of Medicine, Ankara, TUR; 5 Department of Internal Medicine, Lokman Hekim Hospital, Ankara, TUR; 6 Department of Gastroenterology and Hepatology, Ankara Yıldırım Beyazıt University, Faculty of Medicine, Ankara, TUR

**Keywords:** oxidative stress, homeostasis, disulfide, thiol, vitamin d deficiency

## Abstract

Objective: Thiol/disulfide (T/DS) homeostasis represents a promising new approach to evaluate oxidative stress. Therefore, we aimed to examine T/DS homeostasis in vitamin D (VitD)-deficient patients.

Methods: We enrolled 154 patients with VitD deficiency and 154 healthy control subjects in the study. For both groups, native thiol, total thiol, and disulfide values were measured. Additionally, considering the obtained 25-hydroxycholecalciferol [25(OH)D] levels, the patient group was further divided into two subgroups (Group 1: <10 ng/mL, Group 2: 10-20 ng/mL), which were compared in more depth according to the specified parameters.

Results: Values of native thiol, total thiol, and disulfide measured in the combination of Groups 1 and 2, comprising individuals with VitD deficiency, proved to be higher in comparison to the control group with statistical significance (p=0.007, p=0.028, and p<0.001, respectively). When subgroups were considered according to VitD classifications, native thiol and total thiol were again higher in Group 1 in comparison to the values obtained for control subjects (p=0.022; p<0.001). While the total thiol level of Group 2 was higher than that of controls (p<0.001), no difference with statistical significance was obtained in the comparison of disulfide levels among the indviduals of Group 1, Group 2, and the controls (p=0.081).

Conclusion: In this study, among patients with VitD deficiency, we have confirmed that values of native thiol and total thiol were increased, while the T/DS balance was found to have shifted in favor of the thiol level.

## Introduction

It is broadly accepted in the medical literature that inflammation and oxidative stress contribute to the development of chronic diseases including, among others, cardiovascular disease, type 2 diabetes mellitus (DM), and chronic kidney disease (CKD) [1,2]. It is furthermore recognized that vitamin D (VitD) has a crucial role in both bone metabolism and calcium (Ca) homeostasis. Studies have revealed that VitD deficiency raises one’s risk of various chronic metabolic diseases [3,4]. Although the particular mechanisms shaping the relationships that exist between VitD level and these chronic metabolic diseases are not fully understood at this time, VitD levels may potentially contribute to disease risk by affecting oxidative stress and/or inflammation.

Previously obtained data clearly show that, in its biologically active form, VitD has in vitro antioxidant and anti-inflammatory effects [5,6]. Some cross-sectional and interventional research projects have further reported the effect of VitD on oxidative and inflammatory markers in circulation [5,7,8].

Dynamic thiol/disulfide (T/DS) homeostasis constitutes a new method that is recently being used to determine oxidative stress levels [9,10]. As thiols are organosulfur compounds possessing antioxidant functions driven by a variety of mechanisms, it is more likely that dynamic disulfide bonds will be accompanied by oxidative stress. Furthermore, dynamic T/DS homeostasis has been found to have important roles in various stages including protein stabilization, enzyme function regulation, transcription, protection against antioxidants, detoxification, proliferation and growth of cells, and apoptosis. In these diverse biological settings, thiols will be oxidized and formed into disulfide bond structures by the oxidants that are present in the environment.

Meanwhile, the disulfides can be cut down again to thiol structures, which allows the balance to be successfully preserved.

This relationship existing between oxidative stress and VitD has been revealed to date by various different methods. However, studies in the literature that investigate the relationship of dynamic T/DS homeostasis with VitD levels are limited in number [11,12]. Therefore, in the present work, we aim to explore the impact of dynamic T/DS homeostasis for VitD-deficient patients.

## Materials and methods

A total of 308 participants were enrolled in the study including 154 patients with VitD deficiency (121 females, 33 males) as the study group, and 154 healthy subjects (130 females, 24 males) as the controls. Appropriate patients over the age of 18 who were admitted to the internal medicine outpatient clinic in accordance with the exclusion criteria were included in the study. Following overnight fasting, blood was obtained from the antecubital vein. Those obtained samples were subsequently subjected to centrifugation for 10 min at 3000 rpm and thereafter stored until analysis at -80 °C. For all participants, levels of 25(OH)D levels in plasma were obtained based on measurements acquired by liquid chromatography-tandem mass spectrometry method using the Waters Corporation autoanalyzer (Likrom, Istanbul, Turkey). All evaluations were based on a reference range of 25-80 ng/mL for 25(OH)D. Levels of Ca and phosphorus (P) in serum were obtained via measurements conducted with the Advia 2400 Chemistry System (Siemens, Washington, D.C., USA). Those with 25-hydroxycholecalciferol [925(OH)D] levels of <20 ng/mL were defined as VitD deficient and those with levels measured to be >30 ng/mL were considered healthy in this regard. The patient group was further subdivided into two based upon the specific VitD levels of these deficient individuals. Group 1 comprised patients with VitD levels found to be <10 ng/mL, while the individuals of Group 2 had levels of 10-20 ng/mL. In contrast, the control group consisted of healthy subjects with normal 25(OH)D values (n=154). For both of these patient groups (namely Group 1 and Group 2), native thiol, total thiol, and disulfide values were compared to the control group’s values. Any individuals with a history of DM, hypo- or hyperthyroidism, hypertension, liver or lung disease, any malignancies, kidney or coronary heart disease, and rheumatological disease were excluded from the study, as were those who used any medications that would affect oxidative status or who smoked. The participants in the patient group and control group having other known chronic diseases that may affect VitD metabolism were not included. Ethics committee approval was received for this study on 17.02.2016, Approval Number: 26379996/64 and all participants confirmed their informed consent.

Again for all participants, the values of native thiol, total thiol, and disulfide levels were evaluated with the assistance of the fully automated system presented by Erel and Neselioglu, operating based on reductions of dynamic disulfide bonds to functional thiol groups via application of sodium borohydride (NaBH4) [9]. In this process, formaldehyde was utilized with the aim of removing any remaining NaBH4, a step that is necessary in such analyses for prevention of the excessive reduction of 5,5′-dithiobis-2-nitrobenzoic acid (DTNB) and subsequent reduction of the disulfide bonds that are generated on the heels of those particular DTNB reactions. For the quantification of total thiol, Ellman’s reagent was utilized. Native thiol contents were subsequently subtracted from the obtained total thiol values and half of that difference (50%) was taken to reflect the quantity of disulfide bonds. Measurements of amounts of native thiol and disulfide were performed with the help of an automated clinical chemistry analyzer (cobas 501; Roche, Mannheim, Germany) and serum thiol and disulfide values are represented here in µmol/L. The study complies with the Strengthening the Reporting of Observational Studies in Epidemiology (STROBE) guidelines.

Statistical analysis

IBM Statistical Package for Social Sciences (SPSS) version 21.0 (IBM Corp., Armonk, NY, USA) was utilized for all necessary statistical calculations and subsequent analyses. Continuous variables were evaluated in terms of distribution with the application of the Kolmogorov-Smirnov test together with normality graphs. However, not all continuous variables conformed with a normal distribution; as a result, values of median (range) are also given throughout this work. The three groups considered within this work were subjected to comparison using the Kruskal-Wallis test or the Mann-Whitney U test in light of the numbers of groups being considered in particular cases. Chi-square tests were used in conducting investigations of the differences between groups more deeply concerning the relevant categorical variables. Values of p<0.05 were accepted as reflecting statistical significance.

## Results

The present study population comprised a total of 154 patients who were diagnosed as having VitD deficiency together with 154 individuals without VitD deficiency as the control group. The median age [43 (18-74) years vs. 38 (21-67) years] and gender distribution (121 female, 33 male patients vs. 130 female, 24 male patients) of the overall patient group and the controls were similar (p=0.058 and p=0.187). Furthermore, no significant differences were identified between these two main groups for either serum Ca or P level (p>0.05 for both). Serum albumin values were seen to be similar (p=0.803) while erythrocyte sedimentation rate (ESR) was higher in the overall patient sample in comparison to the controls without VitD deficiency (p=0.020). Likewise, the values obtained for native thiol, total thiol, and disulfide for individuals in the overall VitD-deficient group were statistically significantly higher than corresponding levels obtained for control participants (respectively p=0.007, p=0.028, and p<0.001). Demographic and laboratory data for these two main groups are presented in Table 1.

**Table 1:** Demographic and laboratory data of the study groups Ca: Calcium; P: phosphorus; ESR: erythrocyte sedimentation rate. Patients: 25(OH)D levels of <20 ng/mL; Controls: 25(OH)D levels of >30 ng/mL p<0.05 was considered statistically significant.

	Patients (n=154)	Controls (n=154)	p
Age (years)	43 (18-74)	38 (21-67)	0.058
Gender (female/male)	121/33	130/24	0.187
Serum Ca (mg/dL)	9.49 (8.30-10.50)	9.40 (8.50-10.30)	0.137
Serum P (mg/dL)	3.40 (2.30-5.20)	3.41 (2.80-5.60)	0.977
Albumin (g/dL)	4.00 (3.50-4.40)	4.01 (3.80-4.60)	0.803
ESR (mm/h)	16 (2.0-47.0)	15 (2.0-37)	0.020
Native thiol (µmol/L)	502.00 (200.30-645.50)	486.60 (369.70-609.20)	0.007
Total thiol (µmol/L)	542.55 (204.10-686.50)	467.75 (351.90-637.20)	<0.001
Disulfide (µmol/L)	20.25 (1.90-39.0)	18.15 (1.45-40.10)	0.028

The overall VitD-deficient group was further divided into two smaller groups in light of the individuals’ exact 25(OH)D levels, whereby Group 1 comprised subjects with 25(OH)D levels found to be lower than 10 ng/mL and Group 2 contained those with 25(OH)D levels of 10-20 ng/mL. The demographic and laboratory data of both of these smaller patient groups were again compared with the data previously considered for the control group (Table 2). 

**Table 2:** Comparison of the laboratory and demographic data of the vitamin D groups and the control group Ca: Calcium; P: phosphorus; ESR: erythrocyte sedimentation rate. Group 1: 25(OH)D levels of <10 ng/mL; Group 2: 25(OH)D levels of 10-20 ng/mL. p<0.05 was considered statistically significant.

	Group 1 (n=60)	Group 2 (n=94)	Controls (n=154)	p
Age (years)	44 (18-67)	41 (18-74)	38 (21-67)	0.143
Gender (female/male)	(48/12)	(73/21)	(130/24)	0.370
Serum Ca (mg/dL)	9.30 (8.30-10.25)	9.40 (8.88-10.50)	9.40 (8.50-10.30)	0.229
Serum P (mg/dL)	3.40 ( 2.30-4.80)	3.40 (2.60-5.20)	3.41 (2.80-5.60)	0.883
Albumin (g/dL)	4.00 (3.50-4.30)	4.00 (3.60-4.40)	4.01 (3.80-4.60)	0.583
ESR (mm/h)	19 (3-44)	15.50 (2-47)	15 (2-37)	0.071
Native thiol (µmol/L)	513.80 (402.10-590.10)	496.50 (200.30-645.50)	486.60 (369.70-609.20)	0.018
Total thiol (µmol/L)	549.30 (440.70-617.00)	536.10 (204.10-686.50)	467.75 (351.90-637.20)	<0.001
Disulfide (µmol/L)	19.85 (3.05-39.00)	20.60 (1.90-37.00)	18.15 (1.45-40.10)	0.081

Serum Ca, serum P, and ESR values of all three groups considered at this stage were similar (p>0.05 for all). At the same time, the native thiol of Group 1 was seen to be higher compared to the value that was obtained for the non-deficient control group [513.80 (402.10-590.10) µmol/L vs. 486.60 (369.70-609.20) µmol/L] (p=0.022). Meanwhile, significant differences in native thiol levels did not exist between Group 2 and the control group or between Group 1 and Group 2 (p=0.252 and p=0.802). The obtained total thiol levels for Groups 1 and 2, comprising VitD-deficient patients, were both seen to be higher upon a comparison with the healthy control group [549.30 (440.70-617.00) µmol/L vs. 467.75 (351.90-637.20) µmol/L, p<0.001; 536.10 (204.10-686.50) µmol/L vs. 467.75 (351.90-637.20) µmol/L, p<0.001]. At the same time, no significant difference could be identified between Groups 1 and 2, namely the subgroups of VitD-deficient patients, for total thiol levels (p=1.000). Disulfide levels were similar among all three groups considered in this stage of the research (p=0.081).

## Discussion

In the research presented here, we used T/DS homeostasis to assess conditions of oxidative stress and observed that native thiol, total thiol, and disulfide levels were all higher among individuals with VitD deficiency upon a comparison with the individuals in the control group.

Oxidative stress may be succinctly described as an imbalance between reactive oxygen species (ROS) generation and the functioning of antioxidant defenses [13]. The deterioration in the metabolic state triggers formations of free radicals [14]. It is furthermore recognized that ROS engender the oxidation of the thiol groups of sulfur, a process involving both amino acids and disulfide bonds [15]. Thiol groups play an important part in the occurrence of antioxidant processes due to their ability to scavenge ROS as well as other free radicals. Also described as mercaptans, thiols are known to have vital roles in processes of homeostasis in terms of reactions of both reduction and oxidation. Disulfide bonds are spoilable; in other words, they may be turned back into thiol groups, allowing the maintenance of the homeostatic balance existing between the thiols and the disulfide bonds. It has been demonstrated by various researchers that the dynamic T/DS balance takes crucial roles in programmed cell death, mechanisms of cellular transduction, cellular enzymatic activity, antioxidant protection, transcription, and detoxification [16,17]. If this T/DS balance is measured toward the disulfide groups, all of the aforementioned biological processes will be affected in a negative way, with the development of various pathologies of both function and organ structures [18].

T/DS imbalance is known to have the ability to cause assorted diseases, including cancer, DM, or cardiovascular disease [19,20]. Deficiencies of VitD are clinically very important; as a result, this deficiency has been emphasized more frequently in recent years and its relationship with many diseases has been investigated [21,22]. However, the mechanism of action of VitD deficiency in other diseases has not yet been elucidated in full. One possible mechanism is increasing levels of oxidative stress coupled with the impairment of T/DS homeostasis.

Cases of VitD deficiency may be similar to enhanced oxidative stress as seen in rats with type 2 DM, in cases of nutritional rickets, in cases of multiple sclerosis, and also in otherwise healthy children [23-26]. There are studies summarizing the antioxidant functions of VitD in the literature. Being an antioxidant, VitD is able to exert antioxidant protective effects for cultured human endothelial cells and retinal cone cells, with the activation of the protective antioxidant Nrf2-KEAP1 pathway in rats induced with DM [27-29]. The anticancer activities of VitD have also been explained by its antioxidant properties [30]. For example, in a meta-analysis by Zhang et al., it was stated that there may be an inverse relationship between serum vitD levels and the risk of liver cancer [31]. Sardar et al. explained that VitD is an antioxidant because hepatic glutathione levels increase in rats being administered cholecalciferol [32]. According to other researchers who explored intracellular pathways utilized by VitD within samples of cultured human umbilical vein endothelial cells that were subjected to exposure to processes of oxidative stress, it is feasible that VitD might stop endothelial cell death by exerting an influence on the modulating of the interplay that occurs between autophagy and apoptosis together with inhibition of the generation of superoxide anions, with VitD furthermore having mitochondrial functions and an important impact on cell viability, making survival kinases become active alongside the induction of the production of nitric oxide [33].

Limited studies have attempted to assess the relationship between VitD deficiency and T/DS homeostasis. In their study that was conducted with 693 healthy adults, Alvarez et al. showed that levels of 25(OH)D in serum had positive associations with plasma glutathione (GSH, a critical low-molecular-weight intracellular antioxidant) but demonstrated a negative association with cysteine (Cys, a key extracellular antioxidant). They also concluded that the concentration of 25(OH)D had an association with GSH and Cys T/DS redox systems in the plasma of healthy adults [12]. In another study, researchers concluded that severe VitD deficiency in healthy children caused deterioration of T/DS homeostasis together with increased protein oxidation and that this situation could not be successfully treated with external VitD supplementation [11]. In a meta-analysis of randomized controlled trials evaluated by Alhabeeb et al., omega-3 supplementation was shown to increase 25 (OH)D concentration [34].

In the study of Erel and Neselioglu, it was stated that the T/DS balance in the plasma shifts toward the disulfide direction in cases of degenerative diseases including DM, obesity, bronchiolitis, and pneumonia. In contrast, this balance is seen to be shifting toward the thiol in cases of proliferative diseases including colon cancer, bladder cancer, kidney cancer, and multiple myeloma [9]. In our work, the disulfide of the studied individuals with VitD deficiency was measured at levels higher than those seen among participating healthy individuals. Nevertheless, when the patients were grouped according to their VitD levels, no significant differences could be identified to exist between the considered groups for disulfide levels. Furthermore, in patients with VitD deficiencies, especially those with 25(OH)D levels of <10 ng/mL, both the native thiol level and the total thiol level were higher than those measured in healthy individuals. We accordingly determined that T/DS homeostasis shifted toward thiol in patients with VitD deficiencies, as is also the case for patients with proliferative diseases. This suggests the possibility of T/DS homeostasis being useful in evaluating correlations between proliferative disease and VitD.

Conflicting results have been obtained among studies attempting to address the effects of supplementation of VitD on oxidative stress. Although there have been works indicating that VitD supplementation can reduce the oxidative stress seen in cases of type 2 DM, non-alcoholic fatty liver disease, or polycystic ovarian syndrome, and even in asymptomatic individuals, there are also studies reporting that it does not affect oxidative stress [11,24,35-40]. The effects of VitD replacement on T/DS homeostasis were not assessed in our study, but we believe that a study of the changes in T/DS homeostasis after VitD replacement would be useful in understanding relationships that may exist between VitD and oxidative stress.

The present study possesses some limitations worth noting. First, as VitD-insufficient patients were not included in this research, changes in the levels of total thiol, native thiol, and disulfide could not be assessed in that particular group. Second, measurements were not conducted again for the participating patients after VitD was supplemented for them; as a result, the effects of that supplementation on these parameters could not be determined.

## Conclusions

We have successfully demonstrated that levels of native thiol and total thiol increased in a sample of patients with VitD deficiency but levels of disulfide did not change and the T/DS balance shifted in favor of thiol. Our study may give an idea about the pathophysiology of disorders thought to be associated with VitD deficiency. We believe that it would be beneficial to consider the possible relationships between VitD and oxidative stress more deeply by undertaking further comprehensive studies.
